# Supplementary material for: Behavioural Systems Mapping of Solid Waste Management in Kisumu, Kenya, to Understand the Role of Behaviour in a Health and Sustainability Problem
Source: Behav Sci (Basel). 2025 Jan 26;15(2):133. doi: 10.3390/bs15020133 (PMC11851750; doi:10.3390/bs15020133)
Supplement: Supplementary file 1 [file behavsci-15-00133-s001.zip › Supplementary File S1. Interview Guide.pdf]

Supplementary File S1: Interview and focus group discussion guide

Participants

- Ask for as much information about participants as possible beforehand and circulate it beforehand to all focus group participants.
- Ask for homogeneous groups in terms of seniority.
- This is advertised as a 3-hour focus group.
- This interview guide might work well with NGOs Industries, Academics participants who implement projects.
- Residents and government officials might not respond much on the questions asking about project implementation since they hardly implement projects.

Materials and structure

- Prompt cards.
- Flipchart or large paper and markers.
- Two audio recorders per group.
- Try to have breaks or make people get up every 45 minutes or so.

Background points to cover

- Information and consent sheet (e.g. **anonymisation**, can leave the study at any time in interview however comments unable be removed from the overall transcripts). Check if participants have any questions before we start.
- As you know, we are here as part of the CUSSH project, and we are interested in how this project fits within your city's decision-making context.
- You (the focus group participants) are all experts and provide different opinions and experiences. Rules for the focus group: accept your view may differ to others in the group, need to respect others' opinions… neither right or wrong but we want to know where you agree or differ in opinions or understanding as that will better inform the learnings we gather on this subject.
- Explain the structure and themes of the focus group at beginning.

#	Questions	Prompts	Research question themes
**PART 1**			
**Signpost**	**In today's focus group we would like to find out about your experiences on recent projects related to [TOPIC AREA].**		
**Prompt card**	**Introduction**<br>● **Your name and organisation**<br>● **A project you have worked on**<br>● **Focus of the project**<br>● **Your role in the project**		
**1**	To begin we would like to go around the group and ask you to tell us about yourself and a project you have worked on<br><br>Please could you introduce yourself and then tell us about your project / work you do *(for government and residents)*, its focus and your role.<br><br>*Write a one-word name for each project/area of work on the flipchart.*	What do you feel you are responsible for?	Warm up and context of participant's role in decision-making (informal and formal)
**1a**	*If needed, follow up to clarify the focus of the project:*<br><br>To what extent did the project or does your work consider urban sustainability?<br><br>To what extent did / does the project / work you do consider health?<br><br>Is that typical?		Trying to get at the relative importance of e.g. sustainability and health.

#	Questions	Prompts	Research question themes
**2**	Were/are any of these projects that you talked about connected at all?<br><br>If so, could you indicate how they are connected and any organisations linking them? *Ask this question to NGOs, Industries, and CBO. It may not apply to residents.*<br><br>*Ask group to draw and label links on the flip chart to indicate connections*	Did you collaborate with others around the table on any/some/all of these projects?<br><br>Was there something specific about this/these projects as to why you might have collaborated here but not others?<br><br>How did the collaboration evolve over time?<br><br>*Probe to determine decision making process if several projects collaborated towards similar outcome.*<br><br>*Look at transparency: Clarity of project objectives; equal distribution of risks, benefits, costs etc; practicality of the projects, e.g. resource constraints; political acceptability; power etc.*	Same as above and stakeholder dynamics
**2a**	Are there any organisations that are missing on this graphic?<br><br>*Leave it to the respondent to measure the importance of a partner organisation*	Are they usually involved in such projects?	
**Signpost**	**Next we would like to go into more detail about each project to understand the goals of the stakeholder organisations or groups involved.**		

#	Questions	Prompts	Research question themes
**Prompt card**	**Stakeholders and their goals**<br>● **Goals of your organisation**<br>● **Goals of other organisations**		
**3**	*Go through the projects one at a time and write responses on a new flipchart sheet:*<br><br>Regarding the [NAME] project/work, what were the goals of your organisation? Why are these goals important to your organisation? Why? Are these the only goals? What values do your goals reflect?<br><br>What were the goals of the other organisation(s) involved in the project? Why are these goals important to your organisation? Why? Are these the only goals? What values do your goals reflect?<br><br>*Repeat the WHY questions until the interviewee talks about underlying values.*	What are the other organisations and stakeholders seeking to achieve?<br><br>Did any organisations support or object to the proposed project? Why and was there any changes over time?<br><br>Ask more about the organisations if it is not clear, e.g. is that a community group?	Same as above, organisational identities and stakeholder dynamics
**3a**	Are there any tensions between the different organisations' goals/objectives for the project?<br><br>Are there any procedures to resolve disagreements if they arise?	Did your organisation have a different goal for what they wanted to get out of the project?<br><br>Did this create any tension?<br><br>How did you all manage those different priorities?<br><br>Did you have a conflict management team which spearheaded conflict resolution?	Stakeholder dynamics, conflict and power
**3b**	What factors have helped to achieve the goals/objectives of the [NAME] project?	Recently or in the past	Barriers to change

#	Questions	Prompts	Research question themes
**3c**	What factors made it difficult to achieve the goals/objectives of the [NAME] project?	Recently or in the past	Opportunities for change
**4**	Beyond these projects, what are the most important priorities for the city as a whole?<br><br>*Use flipchart paper*<br><br>*Be aware of County Development Plans - briefly:*<br>*Nairobi CDP 2018-2022 priorities: built infrastructure; economic growth opportunities (incl. youth, women, disabilities; affordable healthcare; vocational opportunities; food security; governance, public participation; housing; clean energy, safe drinking water, sustainable waste/sanitary services*<br>*Kisumu CDP 2018-2022: Revitalize agriculture; Ensure a healthy population living in a clean and safe environment; Build modern physical infrastructure; Promote skills development; Conserve the environment + lakefront business; Provide housing; Promote sports, culture/arts; Promote vibrant service sector, supported by Sustainable energy).*	Which are the most important goals to achieve for this city?	Trying to get at the relative importance of e.g. sustainability and health.
**BREAK**			
**PART 2**			

#	Questions	Prompts	Research question themes
**Signpost**	**In the previous part we discussed the goals of the projects you are involved in, and why those goals were important to different stakeholders.**<br><br>**In the next part we would like to think about how those projects moved from goals to actions, and how decisions were made in that process. We would especially like to understand what factors typically influence decisions.**		
**Prompt card**	**Factors that influence decisions**<br>● **Process of making decisions**<br>● **Factors affecting decisions**<br>● **Information used to inform decisions**		
**5**	When moving from goals to actions, what is the process you or others go through to make decisions?	What is the process you go through?<br><br>Who is involved?<br><br>Is this the same or different in different organisations?	Governance and decision-making processes, power<br><br>Policy agendas, governance, decision-making
**6**	What are the factors affecting decisions being made?	i.e. prompt only if need– structures, people, strategies, resources, organisation, practice norms,	Same as above

#	Questions	Prompts	Research question themes
**7**	What information do you and your organisation use to inform decisions?	Where does the information come from?<br><br>Are there alternative policy/development options that were abandoned?<br><br>Were there any assessments of economic/social/environmental impacts that drove the direction?<br><br>Did national or other strategies influence the direction?	Same as above and cultures of evidence
**7a**	*If they mentioned research or evidence:* You mentioned the use of research/evidence; how is it used?<br>In case there were any conflicts, did any groups use some research/evidence to back up their position?<br><br>*If they did not mention the use of evidence:* What is the role of scientific evidence as one of the sources of information?	Try to find out what they actually mean by evidence and research<br><br>To what extent is research used to inform decisions? What types of research? How is it used?	
**7b**	Is scientific evidence ever one of the sources of information? How is it used?<br>Are other kinds of evidence used as sources of information? How are they used?<br><br>How do you access such information? (i.e. Do you 'google search for information, ask a university scientist or an organisation?)	In case there were any conflicts, did any groups use some evidence/information to back up their position?<br><br>Was there open mindedness among partners, i.e. did partners change their positions?	Perceptions of scientific evidence and its role in decision-making

#	Questions	Prompts	Research question themes
**7c**	Do you think information can be used more effectively to inform decisions than it is currently used? How?	Are experts sought to provide specialist knowledge?<br><br>How are they involved in the process and at what time and in what way?	
**BREAK**			
**PART 3**			
**Signpost**	In the previous parts we have discussed goals and decision making in projects related to [TOPIC AREA].<br><br>For the final part we would like to talk about the CUSSH project.<br><br>*Invite participants to read the CUSSH booklet and think about questions or comments. This could be done in pairs.*		
**Prompt card**	**The CUSSH Project**<br>● **Questions and comments**<br>● **Methods and decision-making**<br>● **Sustainability and health goals**		

#	Questions	Prompts	Research question themes
**8**	Do you have any questions or comments about what you have read?<br><br>*Take time to make clarifications and explain aspects of the project in more detail.*<br><br>What are your initial reactions?<br><br>What is similar or different about the CUSSH project and the projects we have been discussing?		Perceptions and goals regarding CUSSH
	What are your initial reactions?<br><br>What is similar or different about the CUSSH project and the projects we have been discussing?		Perceptions and goals regarding CUSSH
**9**	On the CUSSH project we are bringing together multiple methods. Are there any methods or combination of methods that you think could inform decisions in Kisumu?	Thinking back to those projects mentioned earlier – how might these methods have helped?	Explore the CUSSH approach as a method for providing scientific evidence
**10**	The goals of the CUSSH project are to achieve transformative changes in the sustainability and the health of cities.<br><br>What would a transformative change in sustainability look like for Kisumu?<br>What would a transformative change in health look like for Kisumu?<br><br>*Write on flipchart, leaving space to add specific indicators for each*		

#	Questions	Prompts	Research question themes
**10a**	What kinds of changes do you think the CUSSH project could influence?	e.g. changes to stakeholder participation, new local information available<br><br>e.g. achieving something that could not have otherwise happened, thinking differently about urban health and climate	Perceptions of scientific evidence and its role in decision-making
**11**	Reflecting on what you have read and what we have discussed, do you think CUSSH is too ambitious or large-scale to be useful for Kisumu? If yes, in what ways? If no, do you think its ambitions and scale is about right, or do you think we could be more ambitious and larger scale?<br><br>If not ambitious enough, what would make it more ambitious?		Possibility for transformational change, facilitators and barriers
**12**	Finally, we want to ask you about how you would determine a general positive development of Kisumu in the long run. What criteria or indicators could be used to measure a successful development in Kisumu?<br><br>What criteria or indicators could be used to measure a transformative change in sustainability and health in Kisumu?<br><br>*Add to flipchart, next to what these changes would look like*		This question tests whether the response is consistent with what was said above in terms of attention and goals. It provides measures for transformation at the same time.

#	Questions	Prompts	Research question themes
**Signpost**	Thank you very much for all of the discussions today. Soon we are going to draw to a close. Before we do, is there anything else that you would like to talk about?<br><br>*Invite participants to think about their final thoughts and comments then go around the group.*		Wrap-up
