# Supplementary material for: Behavioural Systems Mapping of Solid Waste Management in Kisumu, Kenya, to Understand the Role of Behaviour in a Health and Sustainability Problem
Source: Behav Sci (Basel). 2025 Jan 26;15(2):133. doi: 10.3390/bs15020133 (PMC11851750; doi:10.3390/bs15020133)

Supplementary File S2: Draft behavioural systems maps

Figure S1

Draft behavioural systems map for expert review (round 1)

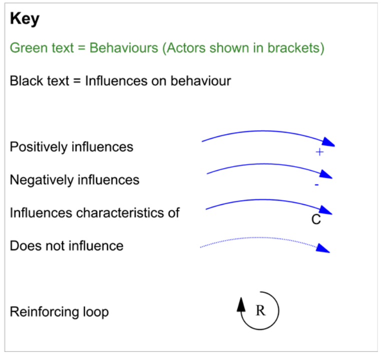

Variables that met the criteria to be included but were not connected to other variables:

Banning plastic bags (National Government)

Cooperation of private waste collectors

Developing circular plastics policy (National Association of Manufacturers)

Distribution of waste infrastructure

Banning plastic bottles (National Government)

Incinerators

Registering private waste collectors (Kisumu County Government)

Biogas digestors

Monitoring and evaluation (Kisumu County Government, Climate Change Directorate, National Association of Manufacturers, Residents associations)

Cultural norms about biogas

Identifying related projects happening in the same area (Kisumu County Government)

Composting

Technical training in biogas (NGOs) → + Installation of biogas equipment (Biogas International)

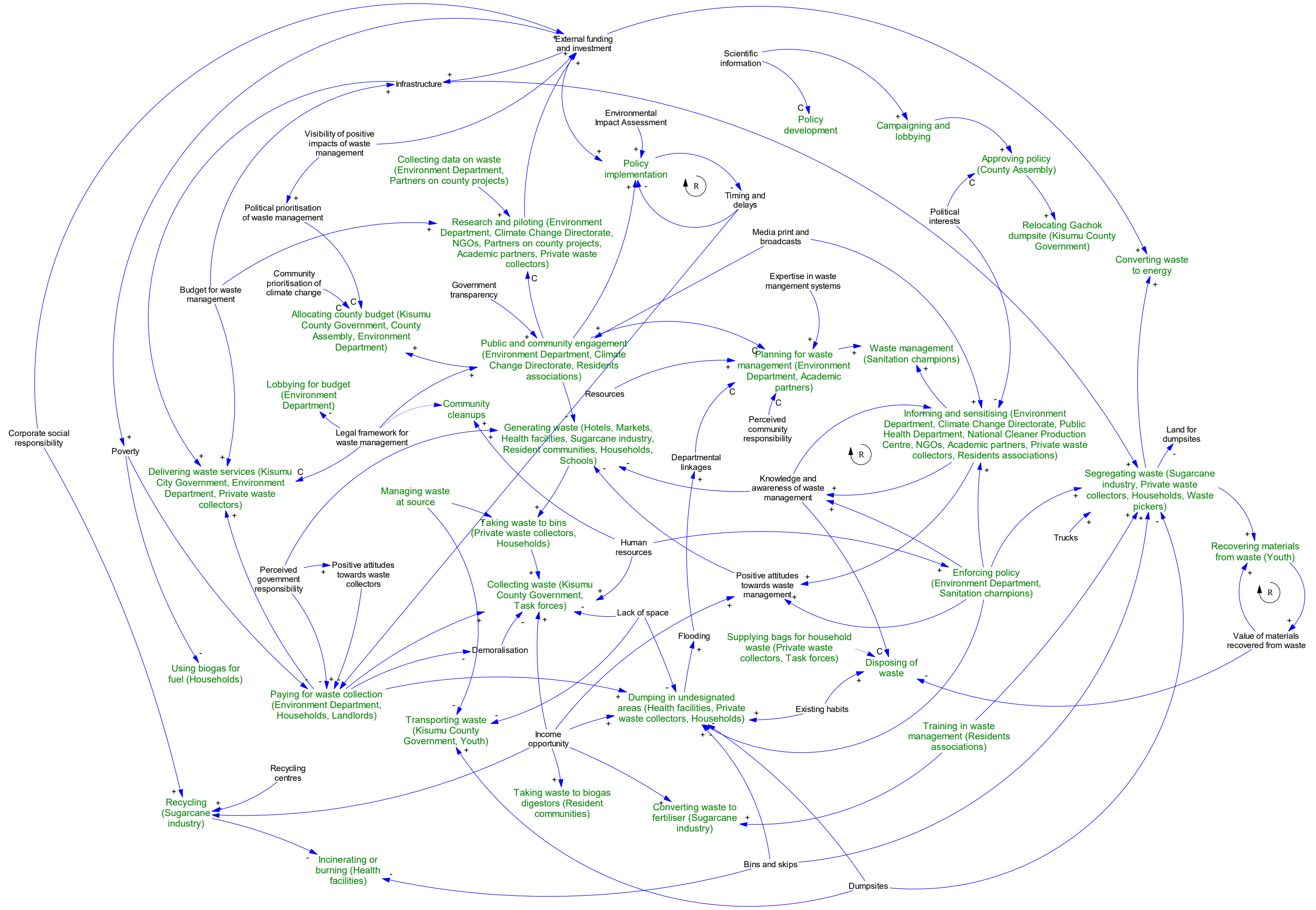

Figure S2

Draft behavioural systems map for expert review (round 2)

Key

Bold text = behaviour
Regular text = influence on behaviour

→ + = Positive causal influence
→ - = Negative causal influence

Actors:

- National Government
- Kisumu County Government (Environment, Water and Climate Change Department)
- County Assembly
- Academic and NGO partners
- Residents Associations
- Waste pickers
- Market traders
- Residents
- Landlords
- Private waste collectors
- Businesses

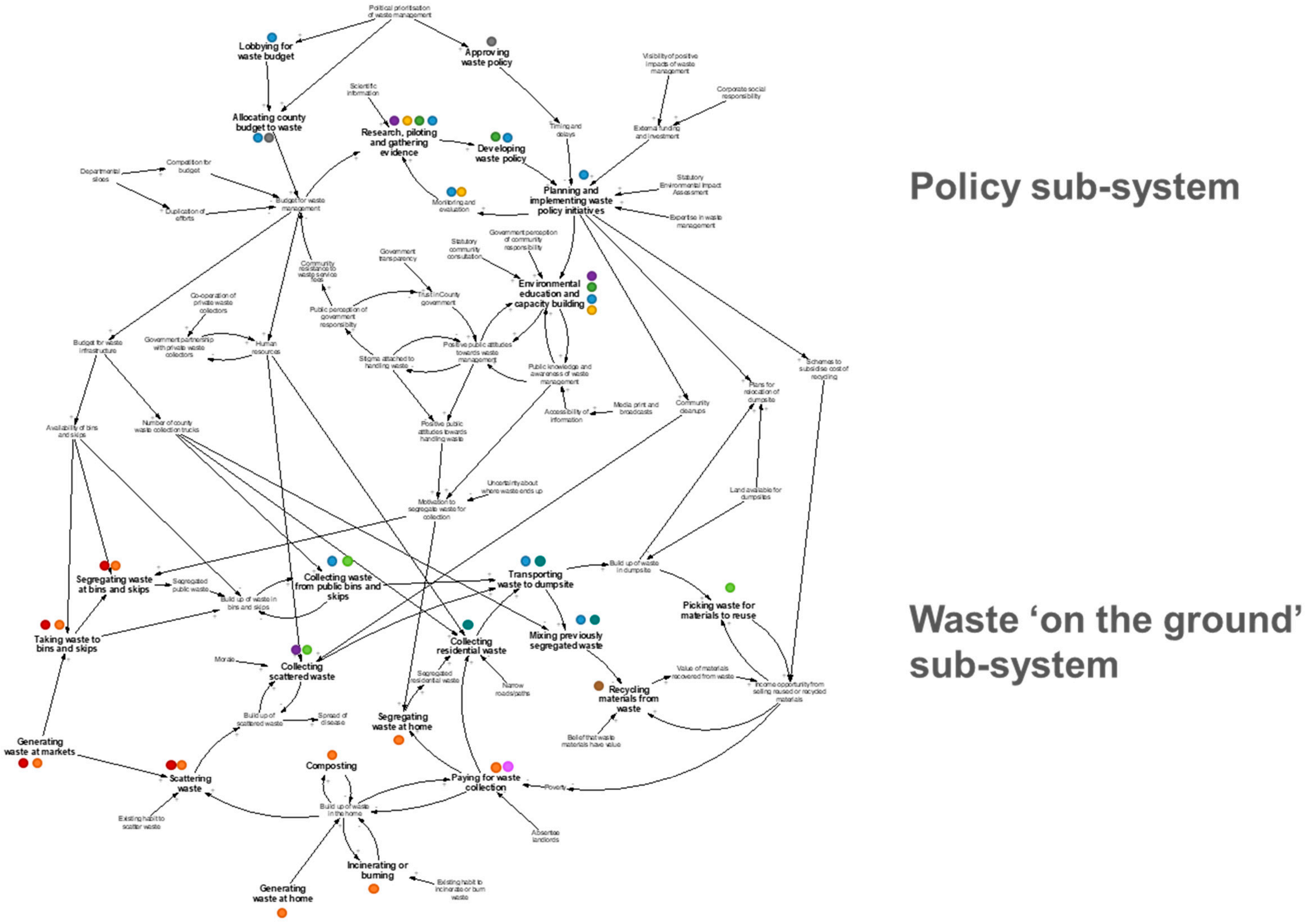

Supplement: Supplementary file 1 [file behavsci-15-00133-s001.zip › Supplementary File S2. Draft Behavioural Systems Maps.pdf]
