# Supplementary material for: Behavioural Systems Mapping of Solid Waste Management in Kisumu, Kenya, to Understand the Role of Behaviour in a Health and Sustainability Problem
Source: Behav Sci (Basel). 2025 Jan 26;15(2):133. doi: 10.3390/bs15020133 (PMC11851750; doi:10.3390/bs15020133)
Supplement: Supplementary file 1 [file behavsci-15-00133-s001.zip › Supplementary File S3. Illustrative Quotes.pdf]

Supplementary File S3: Illustrative quotes

Table S1

Illustrative quotes for behaviours and actors within three sub-systems of waste management in Kisumu.

Sub-system	Behaviour	Actor(s) connected to the behaviour	Illustrative quote(s)
Policy-making	Allocating county budget to waste	County Assembly members, County government employees	"We try and work with our committee members of the assembly because budgets are passed from there" [County Government]<br>"It is the Assembly that apportions resources and they have been giving us little from what we request" [Green Climate Fund workshop]
	Approving waste policy	County Assembly members	"You may have a good plan, you want to do something, maybe in the city, you have a specific area where you want to deliver it then everything has to be subjected to that bureaucratic process of approval." [County Government]<br>"When it comes to the Assembly you find that before they pass a policy, you have to do a lot of lobbying" [County Government]
	Developing waste policy	National government employees, County government employees	"In our directorate, we have meetings and then brainstorm with ideas on what can happen like which development project we should put in place." [County Government]
	Enforcing waste management policy	County government employees, Residents association members	"Sensitization is good but then at the end of the day also the county government, there is a reason why devolution was put in place. I believe the county government should do some little enforcement" [CBOs]<br>"We trained these Champions, they see that their area remains clean. Their area, every area, like Central One we have ten people, Central Two, ten people, Kasarani….like that. So, if you just throw things like this, they take your photo and tomorrow they report you to the County Government." [Residents]
	Gathering evidence	Residents association members, Academics, NGO agents, National government employees, County government employees	"The departments are allowed to do some baseline surveys and they sometimes allocate funding for that. So, they can partner with the university ... there is also feasibility studies which inform the planning and project management" [County Government]<br>"We are piloting in Ahero a cash to trash, zero waste to land field policy. And we are trying to do, we have partnered with the County Government of Kisumu at the moment, they have allocated us an area of land in Ahero market, to do the pilot, to demonstrate its workability." [CBOs]
	Implementing waste initiatives	County government employees	"The current project that is on-going is Kisumu Integrated Sustainable Solid Waste Management … We are implementing that strategy. The component of the strategy includes both doing the infrastructure, the hard infrastructure for waste management and soft infrastructure meaning that we are working with community groups also to supplement what the county is doing e.g. we have registered private waste collectors to supplement the public system handling waste. And all this is in the framework of Kisumu integrated solid waste management plan. Within that framework, one of the challenges that were identified in the solid waste management system was for example, infrastructure for managing waste, trucks for collection, waste bins, management of waste, heavy equipment for the management of the dumpsite. So those are now the components that are being implemented in line with that strategy framework." [County Government]

	Monitoring and evaluating policy initiatives	Academics, NGO agents, County government employees	"Mostly in our line we don't focus on waste directly ... we try see quantity of waste and it like talking to different officers on the ground for Environment Sub-county to get their approximation of the total number of waste they receive or get." [County Government] "We have a major year review where we can…. where we evaluate whatever we have done, and maybe incorporate the new things that have come up during implementation." [County Government]
Public waste management	Burning waste	Residents	"People just manage their waste the way they think is best for them. So, they burn it, just a small pit next to, within the compound and they just burn their waste." [Green Climate Fund workshop]
	Collecting residential waste	Private waste collectors	"The waste collectors, I mean the private, the private waste collectors are very important people, stakeholders in these aspects. Some of them have a proper way of even scheduling their collection either once or twice a week and they know the people, the households where they collect from." [Green Climate Fund workshop] "It is the organic that attracts the cockroaches, the rats and what have you that you want out of the house on a daily basis. So, having a system where you collect your organic everyday would make more sense than collecting once a week for every household." [CBOs]
	Collecting scattered waste	Residents' association members, Waste pickers	"The Directorate of Environment, they are involved in clean-up activities and this is in markets, so they do clean-up activities monthly or weekly depending on the location" [County Government] "It is the CBD which is clean but not the estates we are talking about, like now the slum areas like Kondele, Mbita and that is why I told you that the county can take days before they come to collect the garbage." [Residents]
	Collecting waste from public bins and skips	County government employees, Waste pickers	"You find that the way from the market there is a certain place the people drop there, you will find a very huge heap. The County government do come to collect them, they bring those tanks which are pulled by tractors and they leave them there." [Residents]
	Composting	Residents	"Some can even have small compost yards in their homes, their homesteads that generate that waste, even crop waste" [Industry]
	Generating waste at home	Residents	"The problem actually emanates from us, the home users ourselves. You know if you want to reduce waste, waste has to be reduced from the source. And the source is actually the house." [Academia] "Who is actually generating this waste and where? It is all of us wherever we are living. It starts in our houses, our estates and goes all the way to the markets and it finds its way everywhere." [Residents]
	Generating waste at market stalls	Residents, Market traders	"Estimates from studies reveal that about 60% to 70% of the waste, we generate is organic on average. If you go the markets the percentage is even higher. So, if we are able to deal with it at that market level, then imagine how much will be saved in terms of collecting and transporting it." [County Government] "Myself I think when we are talking of solid waste management, this is a very serious issue. Because it is a contribution of people everywhere day, every day and people are many, people are staying together, they go to the market to buy things, they come with polythene bags and so many things, you see, but they just throw it like that." [Residents]
	Mixing previously segregated waste	Private waste collectors, County government employees	"If you look at the vehicle that collects the garbage that is from the county, they take like three bins which separate the papers, plastic and bottles, but when it comes to collection, it is only one vehicle and now is all mixed waste. So we are trying to do separation at source and then the county are mixing the waste going to the dumpsite" [CBOs]

			"We were looking at a situation where we have different bins labeled, to have organics put separately, inorganic separately and the others. This one has not worked for us so well because of partly us not investing in appropriate trucks and also changing mindset of local people because even if they were to do that, we still also lump these things in the same truck, they go to the same place, so this one beats the whole purpose of recycling waste." [Green Climate Fund workshop]
	Paying for waste collection	Residents, Landlords	"Waste collectors … collect waste in certain estates and they charge at the end of the month." [Green Climate Fund workshop]<br>"Failure by this client to respond to the payment actually that one hinders our services." [CBOs]
	Picking waste for materials to reuse	Waste pickers	"There are some waste pickers who collect these PETs who take them to somebody maybe who does recovery" [CBOs]<br>"Actually, most urchins go to the dumping site to look for metal. So, they will turn it around looking for the metals and take it to the recyclers." [Industry]
	Recycling materials from waste	Business employees	"Recently as a mitigation measure towards, still the menace of that Bagasse as a waste, we have come up with a paper plant, that converts it to paper, brown paper and we use that paper for wrapping materials and envelopes and making boxes" [Industry]
	Scattering waste	Residents, Market traders	"Now when you are in the rural area the tendency is most of the stuff you are using is bio-degradable, you can spread it into your kitchen garden ... What happens when we come to the city? We look for where to put it especially if you live in a small, let us say we call them plots, and there is nowhere to throw it, so you sweep and leave it somewhere on the road for somebody else to take care of." [Academia]<br>"After producing your waste you have to take them somewhere which is set aside for waste collection within a market. And this is what people are not doing. If it is taken there, maybe at the right time, the right people will come for it. And people have an attitude of dropping it everywhere" [County Government]
	Segregating waste at bins and skips	Residents, Market traders	"We have about two or three skips in a location, well labeled. And that is part of the work, because we want to go the hard way. We will have somebody literally standing there and directing people, put this one here put this other one here." [Green Climate Fund workshop]
	Segregating waste at home	Residents	"People were talking about the separation at source, it is just a matter of behavior change. We have tried to give them free bags to separate, when it comes to collection you only collect one bag. If you ask where is the other bag, people have just kept it for clothing or whatever." [CBOs]
	Taking waste to bins and skips	Residents, Market traders	"When we take community cleanup activities to those kinds of neighborhoods, we try and impress upon them that even you as an individual, you as a household, to some small extent this is what you can do for us: get your waste from your household to at least a receptacle then we can come and pick it from there." [County Government]<br>"I live at a place where maybe the receptacle is maybe a kilometer from where I live, the law assumes that it is my responsibility to go to that bin and put that waste."[County Government]
	Transporting waste to dumpsite	Private waste collectors, County government employees	"Muhoroni is a sub county and just one but satellite dumps that you are talking about, they are not very close to town but we bring waste from all those places to dump in the city." [Green Climate Fund workshop]<br>"Most of the waste was coming from the sub counties like Nyakach, Maseno come to the dumpsite here in Kisumu." [CBOs]

Policy-public interface	Public capacity building	Residents association members, Academics, NGO agents, National government employees, County government employees	"We have really done a lot of capacity on that in the community groups and also in schools about the separation of waste and how to recycle them. So, capacity building issues of concern are composting issues and also disposal in terms of separation." [County Government]<br>"So, we realized that whether we give bags, whether we do what, these people still throw these things out. So, we came with education now. We educate them, when we educate them with the Public Health Officers, we are together with the NGOs especially the university, we came with them, some people also came from Nairobi university, we team up with them for education and we did that for almost three months in the community." [Residents]<br>"One of the strategies we have used and fared successfully is doing community cleanup activities where we do clean up in neighborhoods, common commercial investments and then we invite everybody to participate including the private sector. So that then becomes a platform where we are promoting involvement in waste management." [County Government]

Table S2

Illustrative quotes for influences on behaviour categorised to COM-B.

Sub-system	COM-B domain (*N* influences)	Influence on behaviour	Illustrative quote(s)
Policy-making	Capability (1)	Expertise in waste management	"Technological challenges, usually is a problem. You want to implement a project and nobody has the technical knowhow on the machinery so that usually is a problem." [County Government]<br>"For us technical staff, we see these reports, we read them ... but when it comes to now trying to, like create a project around it, it is sort of difficult. mostly it is consuming personally, personal knowledge" [County Government]
	Motivation (6)	Corporate social responsibility	"Apart from just relying on the county budget allocation for that kind of intervention, we reach out ... We go to Safaricom and tell them if you help us install this it will give you visibility that in terms of your Corporate Social Responsibility, Safaricom is investing" [County Government]<br>"We call it mandatory CSR to protect the environment ... The plant is employing a number of people and we also need to protect the environment we are to rely on. So that is why we cannot afford as a plant not to take care of these wastes." [Industry]
		Government belief that the public should participate in waste management	"Traditionally waste management has been the business of the government and now we are trying to get hold of that, because if you look at even the waste management regulations it is very clear that it is the responsibility of the generator of that waste to manage it up to the point provided for the government, which more often is a litter bin when you are walking in the streets or some waste receptacle when you are back in your house." [County Government]<br>"The community has been having an inclination that it is the role of the municipality or the city to make the city clean and therefore they don't care the way brother CS is saying but now we want it to be changed the other way round."[County Government]
		Government sense of responsibility for waste	"Local authority is mandated to do service delivery. And service delivery, one, is solid waste management. Now the population knows that the core activity of the local authority is to evacuate their waste but we are changing the concept by involving CBOs, SMEs. That is a sustainable system from the department of environment." [County Government]<br>"The people believe it is the duty of the government, the responsibility of the government to take care of the waste all over the town. So, the county government as I said earlier must also improve because as things stand now, there is a lot of laxity." [Residents]
		Plans for relocation of dumpsite	"We have one dumpsite and it is within the city. It is not healthy to have it within the city. We have a plan to relocate it maybe to somewhere around the city maybe in the villages around and being these areas are managed by or are manned by politicians, we find it hard to relocate this dumpsite." [County Government]<br>"So, at one point the county government by then it was still a Municipal Council, they wanted to relocate the dumpsite ... at the end of the day the residents were like, no, it is not possible, you cannot have it here. And that was the end of it. So, the project had to stall." [Academia]
		Plans for waste initiatives	"It is a requirement that within every department, you have a strategic plan. That is a policy guide. And within that strategic plan, your reference tools include the national document." [County Government]

			"We also have KISWAMP ... that is the overall strategy that the county intends to use to follow in managing solid waste in Kisumu." [County Government]
		Political prioritisation of waste management	"It is not only the community that politicize the issue of the waste management. Even the political class themselves. You find that a ward representative will come and tell the people you know they are bringing waste to your area, don't accept it. So, any time people go there like to do public participation on bringing the waste there, people will just refuse because they have already been incited by... their representative. So political interference is both, the community and the political class." [County Government]<br>"I am really motivated with our Governor at the moment. At least we have somebody who is really supporting waste management issues wholeheartedly in the name of a governor." [County Government]
	Opportunity (10)	County budget for waste management	"What has been difficult … One is the aspect of funding, that is why we are going for Green Climate Fund. That is an aspect which is very critical." [County Government]
		Delays in receiving resources	"Sometimes, because of the allocation, you find that a financial year has ended like currently, as a department we do not have fuel. So, our truck cannot venture out, so we just have to borrow. So, within that one week of trying to borrow fuel here and there, things are getting messed up in a market somewhere." [Green Climate Fund workshop]<br>"Currently there is this standoff between the National Assembly and Senate over monies supposed to go to the counties, so many things are going to be affected by that because if the counties are not going to receive money soon, meaning delays in project implementation." [County Government]
		Departmental competition	"The county as a whole, what we say is our budget is only this limited, and we have ten departments that are competing for these resources." [County Government]
		External funding for sustainability initiatives	"The priority of our goals is always to attract funding or attract partnership with other people because where we are, challenges are immense" [County Government]<br>"Donor support has been quite important, and in the recent time we have the Kisumu rural project which has a French funded development intervention for in the city that aimed at improving new infrastructure, commercial infrastructure, roads, etc. and through that project we have been able to get quite a bit of equipment that is helping us in waste management." [County Government]
		Government partnership with private waste collectors	"We have some weaknesses, especially in terms of consistency in budgetary support and other issues, and that is why we have allowed the private waste actors to step in to ensure consistency and sustainability" [County Government]
		Human resources	"Staffing levels is very low when it comes to waste management in Kisumu and you will find that we only have one officer per sub county which covers over hundred thousand people or over 20 households so you find that is a challenge and within the city where we have slums and the staffing is very low" [Green Climate Fund workshop]<br>"Another undoing we have had as a county is, we have very few enforcement officers and because of that in as much as you can give technical advice but you will not be everywhere to arrest people and put people in order." [Green Climate Fund workshop]
		Schemes to subsidise cost of recycling	"We are currently working with the county government to identify areas where we can put up collection points to act as incentive for recyclers, so that they don't incur other costs in terms of collecting the PT bottles. Although at the moment we are also giving incentives to the recyclers by setting up a fund which is paid for by the industrialists so that they can use these resources to buy the PT bottles." [Industry]

		Scientific information	"I think scientific evidence is very critical but maybe as a county we have not done much but for our planning purposes ... You see sometimes what inhibits that is the cost of conducting some of these researches." [County Government]
		Statutory Environmental Impact Assessment	"The Environmental Impact Assessment report is a questionnaire within the setup and it has provision of input on the community. So, if you subject it, and approve it and submit it, you will be given a license and without that then the project has some challenges in implementation." [County Government]
		Visibility of positive impacts of waste management	"Managing waste, I think is quicker than managing the slums you know. It is a quicker one and easier to see. It is easy to see and you can easily see the impacts if it is successful." [Academia]<br>"When I clean a market, the traders may have a feeling of that impact. But even then, because it is something recurrent, tomorrow when you come back it is already dirty. So, it doesn't stick in mind." [County Government]
Public waste management	Capability (1)	Uncertainty about where waste ends up	"I have those waste bins and somebody collects them every week and I pay KSh200 monthly to the person that collects the waste from me but there is no tracking document so I don't know where he takes them anyway. I have never followed up." [Industry]
	Motivation (5)	Belief that waste materials have value	"The government or even the private sector is doing little to promote waste entrepreneurship as a source of employment in Kenya right now, but then ... I believe most of the young people would start seeing and believing waste management as a business opportunity among themselves." [CBOs]
		Existing habit to scatter waste	"You see this culture of dumping waste aimlessly waste in the estates and now it has become a culture and it is a routine. You collect waste today, and it becomes clean, you go clean an area today tomorrow you put a warning, tomorrow the same place you will find the dumping has been done." [CBOs]<br>"It is the order of the day. Isn't it? You peel, you eat, you get peels from the food, maybe you are eating a banana; you just throw away the peels without thinking about the environmental issues. It is a habit." [Academia]
		Opportunity to make income from reused or recycled waste	"Our goal is to reduce the amount of waste that goes to the dumpsite by recovery and recycling, and goal number two is to use waste an opportunity for creating employment to our fellow youth." [CBOs]<br>"Most of they are unemployed and when they get this business at least they can get something out of it. ... They collect the plastics then sent to the recycling companies then they can raise revenue for themselves" [County Government]<br>"We do it [recycling] because one, first for purposes of environment compliance and also, we are looking at waste as an income." [Industry]
		Public motivation to segregate waste for collection	"If you tell me about sorting and I don't understand the value of sorting, tell me sort because where these things are being taken to, they will translate to business. But for me, there is no motivation in itself, and that is where we are stuck." [County Government]
		Sense of ownership of public space	"People are not so much aware, of what is theirs. For example, is you could go to the lake and ask who owns the lake, you realize that some of us do not know that the lake belongs to them? Some will say it belongs to what, some will say the county government, some will say the national government and so those are the challenges we are facing" [CBOs]
	Opportunity (15)	Absenteeism among landlords	"Actually, what makes it difficult it is absentee landlords. You find that a landlord is someone who is absentee, maybe staying in a rural area, so it is the agent who is now taking the contract. But the agent doesn't stay there, his purpose is just to collect money and remits to the landlord ... So we strike a deal with the landlord or an agent that all rental fee to be included like garbage collection, and even sanitation maintenance." [CBOs]
		Availability of bins and skips	"After generating this waste in my house is there any place that is designated closer to where I am living, where I can take my waste then? Or must I go all the way seven kilometers where Gachok dumpsite is located?"

			[Residents]<br>"There are bins but most of these bins we are having currently within the city, are dilapidated and there is a plan to replace them." [Green Climate Fund workshop]
		Availability of receptacles	"I even tried to give an household one apartment three containers, you find that they are going to use one container, the agent or the landlord kept the other container for water storage." [CBOs]<br>"If you can properly train the landlords and give the different bins separately in each and every household, that will curb the issue of waste segregation." [CBOs]
		Build up of scattered waste	"At the evening, wee hours, at seven in the evening or eight you will see women, ladies, with bags, not just one, they accumulate maybe weekly, then you see the big bags in the dark going there. So, at the end of the day that place has become, it is becoming a small dumpsite" [Green Climate Fund workshop]
		Build up of waste at markets	You find that a way from the market there is a certain place the people drop there, you will find a very huge heap. The County government do come to collect them, they bring those tanks which are pulled by tractors and they leave them there. But sometimes they forget to come and collect them. You find the heap is now full and full and everything is now strewn all over." [Residents]<br>"There is some waste that even within that market, they have to be evacuated like hourly. Like when you consider waste from the vegetable vendors and even the fishmongers. They have to be taken care of like hourly but you see what was designed there, it was meant for fresh produce like fruits, clothes and the rest which can be taken care of maybe after two day, you come and evacuate them after two days." [County Government]
		Build up of waste in bins and skips	"We are trying to be very careful with skips because we are discovering that in any location that we put a skip, if not well done and thought of, we turn it into a dumping site." [Green Climate Fund workshop]
		Build up of waste in dumpsite	None[a]
		Build up of waste in the home	None[a]
		Number of waste collection trucks	"So, we have two trucks that are delegated to work out in the sub-county and we have three that are working within the city. Although sometime this number vary because of breakdown like currently we only have one that ventures outside to collect waste." [Green Climate Fund workshop]<br>"The government should also increase their capacity in garbage collection. If it is about trucks, if it is about manpower, they should look into that." [Residents]
		Poverty	"The rate of poverty is very high. You see at times, even per day, he or she doesn't have the money to have even two meals. He can have only one meal with the children. So, how can he or she pay for the garbage?" [Residents]<br>"There is no private collectors who might feel interested in serving the low income. The low income will not even manage to pay Ksh 50 for waste collection, that is why you find that they are the ones who are more marginalized." [CBOs]
		Segregated public waste	None[a]
		Segregated residential waste	None[a]
		Spread of disease	"You see when the rain comes you see many drainages blocked. Why? Because they just throw everything in the drainages. And that one also makes the County and even to be coming again and again picking up garbage and stopping the water to flow. And during rains you see there is a lot. There is a lot of drainages blocked and

			you see there can be some airborne diseases" [Residents]<br>"They forget to come and collect the waste and you know when that one goes out of hand it can even cause a health hazard" [Residents]
		Value of materials recovered from waste	"We are encouraging private entrepreneurs to get in to the waste sector because there is big business there, and the business comes when this waste is managed in a particular way from that point of generation. If I am in my house and segregate the waste, I can get certain value of that waste." [County Government]
		Width of roads	"In informal settlement … you find that moving a truck between those houses is impossible. Getting enough personnel to be able to move in between those houses to pick that waste is almost practically impossible." [County Government]
Policy-public interface	Capability (1)	Public knowledge and awareness about participating in waste management	"I think public awareness campaigns should be enhanced by the stakeholders ... the people must be told or must be reminded that just how to manage the waste: you don't have to throw anything anywhere - so that the people become part of the process right from where they live in the slums to the town, everywhere. People should be made to understand the importance of clean environment." [Residents]<br>"I think it all boils down to sensitization of people through very many sources and so on how to dispose different categories of waste." [Industry]
	Motivation (5)	Positivity of attitudes about participating in waste management	"I think people have negative attitude towards waste to an extent that any material they are using for example this one, after use, it becomes something that you don't want to associate with." [County Government]
		Public belief that waste management is government's responsibility	"The attitude towards garbage collection to the residents, they assume that is the local government's responsibility ... they feel they pay taxes to the local government so that the local government should give them the services" [CBOs]
		Public motivation to participate in waste management	"I have even seen a case where you find waste scattered in some place and you ask the traders who did this and they tell you they don't know. It was done at night. It doesn't bother them, what they want is for you to clean it up ... The people who do that do not really care much as to how we can in a sustainable way sort this problem." [County Government]
		Public sense of responsibility for waste	"So, they say you need to know ten houses surrounding you and the nyumba kumi initiative issue is to do with security purposes. And it I think if we can borrow that concept on the issue of waste management so that for example, she is my neighbor, I want to know where does she throw her waste. She should also do the same to another person and then to another person then I think management of waste will be a self responsibility." [Residents]
		Public trust in County Government	"How do we trust the county government that really they can now really help this community to work? Because we are paying the taxes." [Residents]<br>"I think we need a lot of trust … my view is that the county government must seriously be on board and they must demonstrate that they are willing to cooperate and partner with the community and serious partnership." [Residents]
	Opportunity (5)	Accessibility of information	"A lot of the community members said that previously they were not involved in some of the decisions made by then the Municipality ... So, the issue was accessing the information but not just accessing information but there was limited information that was shared with them. And also, there was the issue of the information they got to hear about was too technical for them. And people just tell them you will not understand this." [Academia]

		Community resistance to waste service fees	"Trying to look at levying fees for waste management services is really a challenge especially in informal settlements and at the household level. Because people feel we are taxed enough to provide all the services, so why introduce another levy that we need to pay for a service?" [County Government]
		Government transparency	"Nobody in the community does even know when the budget is coming or what. How the county is running they don’t know. Just very few people because at the time of public participation they don’t see what the government is doing."[Residents]<br>"Transparency and accountability to the county government should also be focused on, because you agree with them on something and then on the day of implementation, they do this and this and that." [Residents]
		Statutory community consultation	"It is anchored in our Constitution where it is mandatory public participation … it is a constitutional right for public participation and we cannot rule out their participation on our implementation of the project." [County Government]<br>"Let us answer that question with the public participation. What is it about? I still believe that bringing one thousand people for a particular forum is still not effective public participation. We need a structured public participation in this where in Obunga, the churches in Obunga, the youth in Obunga, people with disabilities in Obunga and all these other institutes and NGOs in Obunga are called to a meeting in Obunga. We have the church, teachers, and all those. Don’t go like the County government does, they tell people there is money, money you are going to be given, KSh 200, soft drink and water. So, people arrive in the bus park and fill then a photograph is taken and publicity is not really there, or the spirit of public participation so that ought to be corrected. But can we have a structured engagement in terms of public participation?" [Residents]<br>"These days people see it like you go out there and mobilize people and you call it public participation, they see it like you are wasting their time, because after that public participation anything that they will pass there, will not come out. It will not be implemented." [Residents]
		Stigma attached to handling waste	"People still believe that waste is actually compared to a bad thing, an ugly thing, things like that so whoever does that [collects waste] actually, they don’t take you as somebody." [CBOs]<br>"They think these are just people for takataka [rubbish collectors] … people are using them like warthogs … We need to change that if we need to move so that whoever is doing this work should be the first person that we look at his life, his rights, his freedom, his welfare." [Residents]

[a] These variables were logically identified as influences on behaviour during the mapping process and were not linked to illustrative quotes in the transcripts.
